# Are these crystals isostructural? Symmetry requirements, extent of difference, and likeness of supramolecular interactions

**DOI:** 10.1063/4.0000980

**Published:** 2025-10-27

**Authors:** Petra Bombicz

**Affiliations:** Centre for Structural Science, HUN-REN Research Centre for Natural Sciences, Budapest, Budapest, Hungary

## Abstract

The intentional design of crystalline materials, the fine-tuning of physical and chemical properties by slight chemical alterations - crystal engineering - requires understanding of solid-state assembly including supramolecular interactions and isostructurality. The balance of spatial requirements and electrostatic effects ultimately determines the molecular arrangement. In isostructural crystals, both the placement of the molecules and the conformation of flexible molecules may adjust to the chemical and supramolecular features. A given packing motif may tolerate small molecular changes, and the structures remain isostructural within a limit despite chemical changes. The investigation of isostructurality leads to a deeper understanding of the close packing principles, the role of molecular conformation, supramolecular interactions and symmetries, in order to be able to perform the directed manipulation of the molecular packing arrangements.

The prerequisites of isostructurality are similar composition and conformation of the compounds, with analogous molecular arrangement in the crystals. Isostructurality calculations and statistical analyses are efficient tools for the discovery and numerical description of isostructural crystals which is not necessarily straightforward [1].

The presented series of compounds (Figure 1) opens up three questions on isostructurality. Are the corresponding structures required to have the same stoichiometry, space group, Z’ and the same symmetry elements? There is no agreement on these issues. How large can the extent of difference be between the corresponding crystal structures that we may still consider them as being isostructural? Can we consider crystals whose cell parameters are similar, space groups are the same and arrangements of the molecules are analogous, the only difference is in the system of intermolecular interactions to be isostructural?

The definition of isostructurality is not explicit about these issues, it deserves reconsideration regarding symmetry, measure of similarity, and formation of supramolecular interactions [2].
